# The Chinese herb component salvianolic acid B induces copper-mediated reactive oxygen species generation and oxidative DNA damage

**DOI:** 10.1186/s41021-025-00344-w

**Published:** 2025-10-30

**Authors:** Hatasu Kobayashi, Kiyoshi Fukuhara, Akiko Ohno, Yuichiro Hirao, Hiroshi Katoh, Yurie Mori, Shosuke Kawanishi, Mariko Murata, Shinji Oikawa

**Affiliations:** 1 Department of Environmental and Molecular Medicine, Mie University Graduate School of Medicine, Edobashi 2-174, Tsu, Mie 514-8507 Japan; 2 Division of Organic and Medicinal Chemistry, School of Pharmacy, Showa Medical University, Tokyo, 142-8555 Japan; 3 Division of Genome Safety Science, Center for Biological Safety & Research, National Institute of Health Sciences, Kawasaki, Kanagawa 210-9501 Japan; 4 Mie Prefectural College of Nursing, Tsu, Mie 514-0116 Japan; 5 Division of Plant Functional Genomics, Advanced Science Research Promotion Center, Organization for Research Initiative and Promotion, Mie University, Tsu, Mie 514-8507 Japan; 6 Faculty of Pharmaceutical Science, Suzuka University of Medical Science, Suzuka, Mie 513-8670 Japan; 7 Graduate School of Health Science, Suzuka University of Medical Science, Suzuka, Mie 513-8670 Japan

**Keywords:** Salvianolic acid B, Danshen, Polyphenol, Copper, NADH, Reactive oxygen species, DNA damage, 8-oxo-7,8-dihydro-2′-deoxyguanosine

## Abstract

**Background:**

Salvianolic acid B (Sal B), a natural polyphenol with potential therapeutic applications, has been reported to induce reactive oxygen species (ROS) generation. However, its underlying mechanism has not yet been fully elucidated. In this study, we investigated copper-mediated oxidative DNA damage induced by Sal B.

**Results:**

Sal B significantly increased the level of 8-oxo-7,8-dihydro-2′-deoxyguanosine (8-oxodG) in HL-60 cells, but not in H_2_O_2_-resistant HP100 cells. The formation of 8-oxodG was inhibited by a Cu(I)-specific chelator. These results suggested that Cu(I) and H_2_O_2_ play critical roles in this process. In calf thymus DNA, Sal B induced 8-oxodG formation in the presence of Cu(II), which was markedly enhanced in the presence of NADH. Using ^32^P-5′-end-labeled DNA fragments, we showed that treatment with Sal B in combination with Cu(II) and NADH caused DNA strand breaks and site-specific base modification, especially at thymine and cytosine residues. These results suggest the involvement of ROS other than •OH and this was further supported by radical scavenger experiments. Furthermore, theoretical calculation data suggest that one of the catechol groups in Sal B is electron-donating. Collectively, these results indicate that Cu(II)-mediated autoxidation of the catechol group in Sal B generates Cu(I) and H_2_O_2_, likely leading to a Cu(I)-hydroperoxide complex formation and resultant oxidative DNA damage. NADH enhances ROS production and oxidative DNA damage by reducing oxidized Sal B and promoting its recycling.

**Conclusions:**

The potential pro-oxidant risk of Sal B should be carefully evaluated when used as a therapeutic agent.

**Supplementary Information:**

The online version contains supplementary material available at 10.1186/s41021-025-00344-w.

## Introduction

Salvianolic acid B (Sal B), a natural polyphenol, is one of the major active constituents of *Salvia miltiorrhiza* (Danshen), which has been widely used as a traditional Chinese herb for the treatment of various diseases [1]. Sal B possesses antioxidant, anti-apoptotic, anti-inflammatory, and anti-fibrotic properties and exerts protective effects on various organs and tissues such as the heart, liver, kidney, lung, and skin [2–4]. Therefore, Sal B has attracted increasing attention as a potential therapeutic agent.

We have previously evaluated the pro-oxidant activities of natural polyphenols for safe therapeutic use and shown that considerable numbers of antioxidants lead to the generation of reactive oxygen species (ROS) [5, 6]. Hydroxyl groups in the phenolic ring of polyphenols can readily participate in redox reactions [7], which are related not only to antioxidant but also to pro-oxidant properties. Sal B has been reported to elevate the levels of ROS in cell models [8–10]. However, its relevant underlying mechanisms remain poorly understood.

Metal ions contribute to the induction of oxidative stress and subsequent DNA damage [11–13]. Several studies report that copper ions promote the pro-oxidant activities of other agents [11, 14–16]. Copper ions tightly bind to DNA, where they can interact with chemical agents and cause oxidative damage to nucleic acids [14]. Our previous work has shown that several polyphenols cause oxidative DNA damage through redox reaction of copper ions [6, 17–19].

In this study, to examine copper-mediated oxidative DNA damage induced by Sal B, we measured the formation of 8-oxo-7,8-dihydro-2′-deoxyguanosine (8-oxodG), an indicator of DNA oxidative damage [20], in cells treated with Sal B in the presence or absence of a Cu(I) chelator. Sal B-induced 8-oxodG formation in calf thymus DNA was also examined in the presence of Cu(II). In addition, to further investigate the underlying mechanism, we investigated DNA damage induced by Sal B in the presence of Cu(II) and its site-specificity using ^32^P-5′-end-labeled DNA fragments.

## Materials and methods

### Materials

Sal B (purity: more than 98%) was purchased from ChemFaces (Wuhan, China). NADH, catalase (30,000 units/mg from bovine liver), 3-(methylthio) propionaldehyde (methional), and bacterial alkaline phosphatase (from *Escherichia coli*) were purchased from Sigma‒Aldrich Co., LLC. (St. Louis, MO, USA). Nuclease P_1_ (500 units/vial) and piperidine were purchased from FUJIFILM Wako Pure Chemical Co., Ltd. (Osaka, Japan). Calf intestinal phosphatase (500 units/vial) was purchased from Roche Diagnostics GmbH (Mannheim, Germany). Diethylenetriamine-N,N,N’,N”,N”-pentaacetic acid (DTPA) and bathocuproine disulfonic acid were purchased from Dojindo Laboratories (Kumamoto, Japan). T_4_ polynucleotide kinase, *Eco*RI, *Pst*I, and *Ava*I were purchased from New England Biolabs Ltd. (Ipswich, MA, USA). *Bss*HII was purchased from Takara Bio Inc. (Shiga, Japan). [γ-^32^P] ATP (222 TBq/mmol) was purchased from Perkin Elmer, Inc. (Waltham, MA, USA). Copper(II) chloride dihydrate (CuCl_2_·2H_2_O), ethanol, mannitol, and sodium formate were purchased from Nacalai Tesque (Kyoto, Japan).

### Measurement of 8-oxodG formation in HL-60 and HP100 cells

The human leukemia HL-60 cell line and its H_2_O_2_-resistant clone HP100 were obtained from the RIKEN BioResource Research Center (HL-60: RCB3683, HP100: RCB0769). The cells were grown in RPMI 1640 medium supplemented with 10% fetal bovine serum at 37 ℃ in 5% CO_2_. HL-60 and HP100 cells (5 × 10^6^) were incubated with Sal B at 37 ℃ for 16 h and immediately washed twice with PBS. The cells were pre-incubated with 200 µM bathocuproine for 30 min before Sal B treatment where indicated. DNA was extracted using a DNA Extractor WB Kit (FUJIFILM Wako Pure Chemical Industries, Ltd.). The DNA was digested into nucleosides with nuclease P_1_ and bacterial alkaline phosphatase. The amount of 8-oxodG formed was measured using a high-performance liquid chromatography (HPLC) system (SLC-10Avp, LC-20AD, SPD-10AVvp, Shimadzu Corp., Kyoto, Japan) equipped with an electrochemical detector (ECD; HTEC-510, Eicom, Kyoto, Japan) as previously described [19, 21].

### Measurement of 8-oxodG formation in calf thymus DNA

Reaction mixtures containing 100 µM/base calf thymus DNA, 20 µM CuCl_2_, and Sal B were prepared in 4 mM sodium phosphate buffer (pH 7.8) containing 5 µM DTPA. The reaction mixtures were incubated with or without 100 µM NADH at 37 ℃ for 16 h. After ethanol precipitation, the DNA was incubated with nuclease P_1_, followed by the calf intestinal phosphatase. The formation of 8-oxodG was measured as described above.

### Preparation of ^32^P-5′-end-labeled DNA fragments

DNA fragments of the c-Ha-*ras*-1 protooncogene [22] and the human *p16* tumor suppressor gene [23] were used for ^32^P-labeling. The DNA was dephosphorylated using calf intestinal phosphatase, and then phosphorylated with [γ-^32^P] ATP and T_4_ polynucleotide kinase. The c-Ha-*ras*-1 fragment was prepared from plasmid pbcNI carrying a 6.6-kb *Bam*HI chromosomal DNA restriction fragment [24]. The ^32^P-5′-end-labeled 435-bp fragment was digested with *Pst*I to obtain a 337-bp (*Pst*I 2345-*Ava*I* 2681) and a 98-bp c-Ha-*ras*-1 fragment (*Ava*I* 2247–*Pst*I 2344). The DNA fragment containing exon 2 of the human *p16* tumor suppressor gene was obtained as previously described [25]. The ^32^P-5′-end-labeled 460-bp fragment (*Eco*RI* 9481-*Eco*RI* 9940) containing exon 2 was further digested with *Bss*HII to obtain a singly labeled 309-bp fragment (*Eco*RI* 9481–*Bss*HII 9789) and a 147-bp fragment (*Bss*HII 9794–*Eco*RI* 9940). The asterisk indicates ^32^P-labeling.

### Analysis of damage to ^32^P-5′-end-labeled DNA fragments

Reaction mixtures containing ^32^P-5′-end-labeled DNA fragments, 20 µM/base calf thymus DNA, 20 µM CuCl_2_, 100 µM NADH, and Sal B in 10 mM sodium phosphate buffer (pH 7.8) containing 5 µM DTPA were prepared. When examining the effects of ROS scavengers and bathocuproine, these reagents were added before Sal B. After incubation at 37℃ for 16 h, the DNA fragments were heated in 10% piperidine at 90 ℃ for 20 min, and were then subjected to gel electrophoresis on an 8% polyacrylamide/8 M urea gel. An autoradiogram was obtained by exposing an X-ray film (FUJIFILM Corp., Tokyo, Japan) to the gel as previously described [26, 27].

Preferred cleavage sites were determined by direct comparison of the oligonucleotide positions with those produced by the chemical reactions of the Maxam-Gilbert method [28] using a DNA-sequencing system (LKB 2010 Macrophor, LKB Pharmacia Biotechnology Inc., Uppsala, Sweden). The autoradiogram was obtained by exposing an imaging plate (BAS-MS2040, FUJIFILM Corp.) to the gel. The relative amounts of oligonucleotides in the treated DNA fragments were measured using a laser scanner (Typhoon FLA-9500, GE Healthcare, Chalfont St Giles, UK) and analyzed using Image Quant TL software (GE Healthcare).

### Density functional theory (DFT) calculations

DFT calculations were carried out to investigate the most stable conformation and the distribution of the highest occupied molecular orbital (HOMO). The calculations were performed using the ωB97X-D exchange-correlation functional with the 6-31G(d) basis set, as implemented in the Spartan 24 software package (Wavefunction Inc., Irvine, CA, USA).

### Statistical analysis

The results are presented as the means ± standard deviations (SD). Differences were analyzed using Student’s t test. *P*-values < 0.05 were considered to indicate statistical significance.

## Results

### Sal B-induced 8-oxodG formation in HL-60 and HP100 cells

**Fig. 1:**
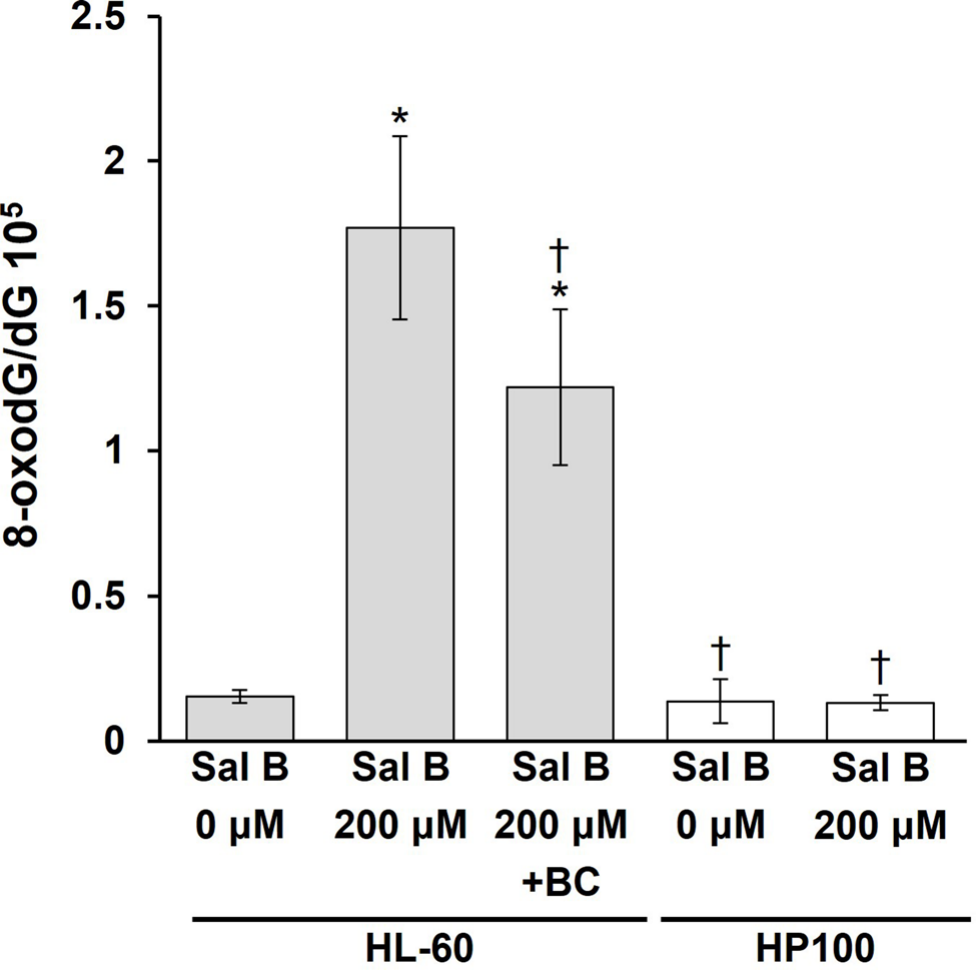
Formation of 8-oxodG in HL-60 and HP100 cells induced by Sal B. HL-60 and HP100 cells (HL-60-derived H_2_O_2_-resistant clone) were treated with 200 µM Sal B at 37 °C for 16 h. Where indicated, cells were pre-treated with 200 µM bathocuproine (BC) for 30 min. After DNA extraction, the DNA was digested to nucleosides with nuclease P_1_ and bacterial alkaline phosphatase, then analyzed using an HPLC-ECD system. *p < 0.05 vs. Sal B 0 µM HL-60. † *p* < 0.05 vs. Sal B 200 µM HL-60. Statistical significance was determined using Student’s t test (*n* = 3–7)

To examine the intracellular oxidative DNA damage induced by Sal B, we analyzed 8-oxodG, an oxidative DNA damage marker [20], in HL-60 cells. Sal B significantly increased the level of 8-oxodG in HL-60 cells (Fig. 1). Pre-treatment with bathocuproine, a Cu(I)-specific chelator [29], significantly inhibited Sal B-induced 8-oxodG formation (Fig. 1). We also examined 8-oxodG formation in HP100 cells, in which catalase activity and resistance to H_2_O_2_ is approximately 18- and 340-fold greater, respectively, than in HL-60 cells [30]. In HP100 cells, no significant increase in 8-oxodG was observed, unlike in HL-60 cells (Fig. 1). These results indicate that Cu(I) and H_2_O_2_ are involved in Sal B-induced intracellular oxidative DNA damage.

### Sal B-induced 8-oxodG formation in calf thymus DNA

**Fig. 2:**
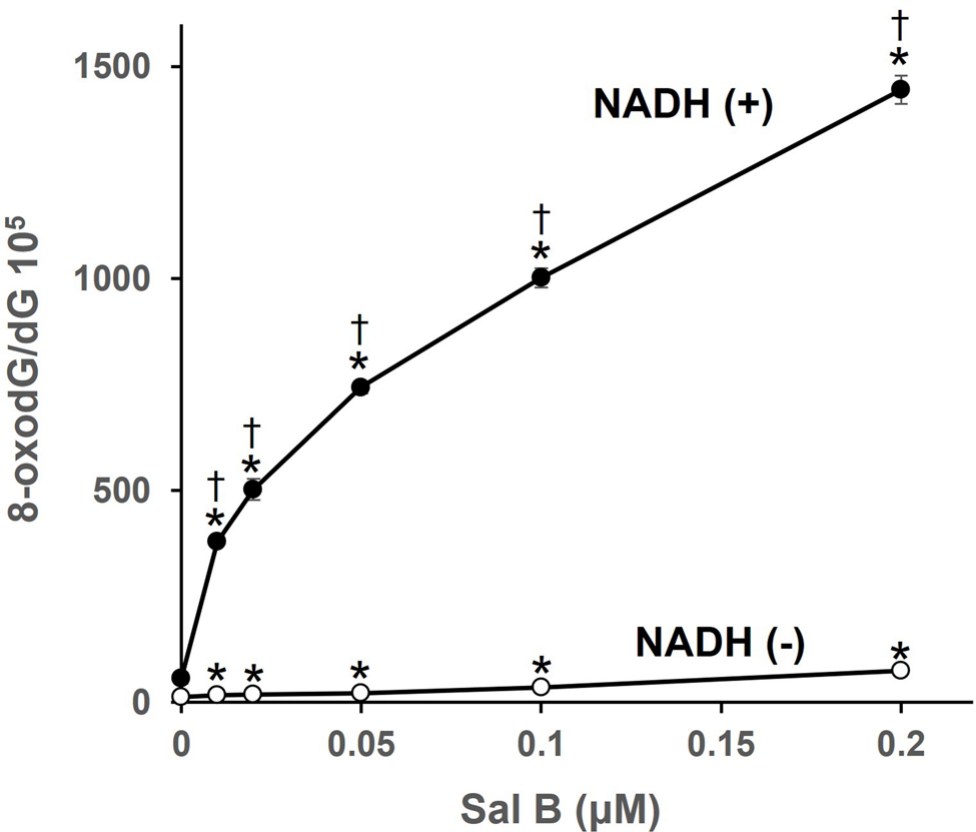
Formation of 8-oxodG in calf thymus DNA induced by Sal B and Cu(II), with or without NADH. Reaction mixtures contained 100 µM/base calf thymus DNA, 20 µM CuCl_2_, 100 µM NADH, and indicated concentrations of Sal B in 400 µL of 4 mM sodium phosphate buffer (pH 7.8) containing 5 µM DTPA. Reaction mixtures were incubated at 37 °C for 16 h. After ethanol precipitation, the DNA was digested to nucleosides with nuclease P_1_ and calf intestine phosphatase, then analyzed with an HPLC-ECD system. *p < 0.05 vs. 0 µM. † *p* < 0.05 vs. NADH(-). Statistical significance was determined using Student’s t test (*n* = 3)

To confirm the induction of copper-mediated oxidative DNA damage by Sal B observed in the HL-60 and HP100 cell models, we further investigated Sal B-induced 8-oxodG formation in calf thymus DNA in the presence of Cu(II). Treatment with Sal B and Cu(II) led to a significant and concentration-dependent increase in 8-oxodG in calf thymus DNA (Fig. 2). Interestingly, the addition of NADH, which can act as a nonenzymatic reductase [31], significantly enhanced the 8-oxodG formation, with the amount of 8-oxodG being approximately 20–30 times greater (Fig. 2).

### Damage to ^32^P-labeled DNA fragments induced by Sal B in the presence of Cu(II) and NADH

**Fig. 3:**
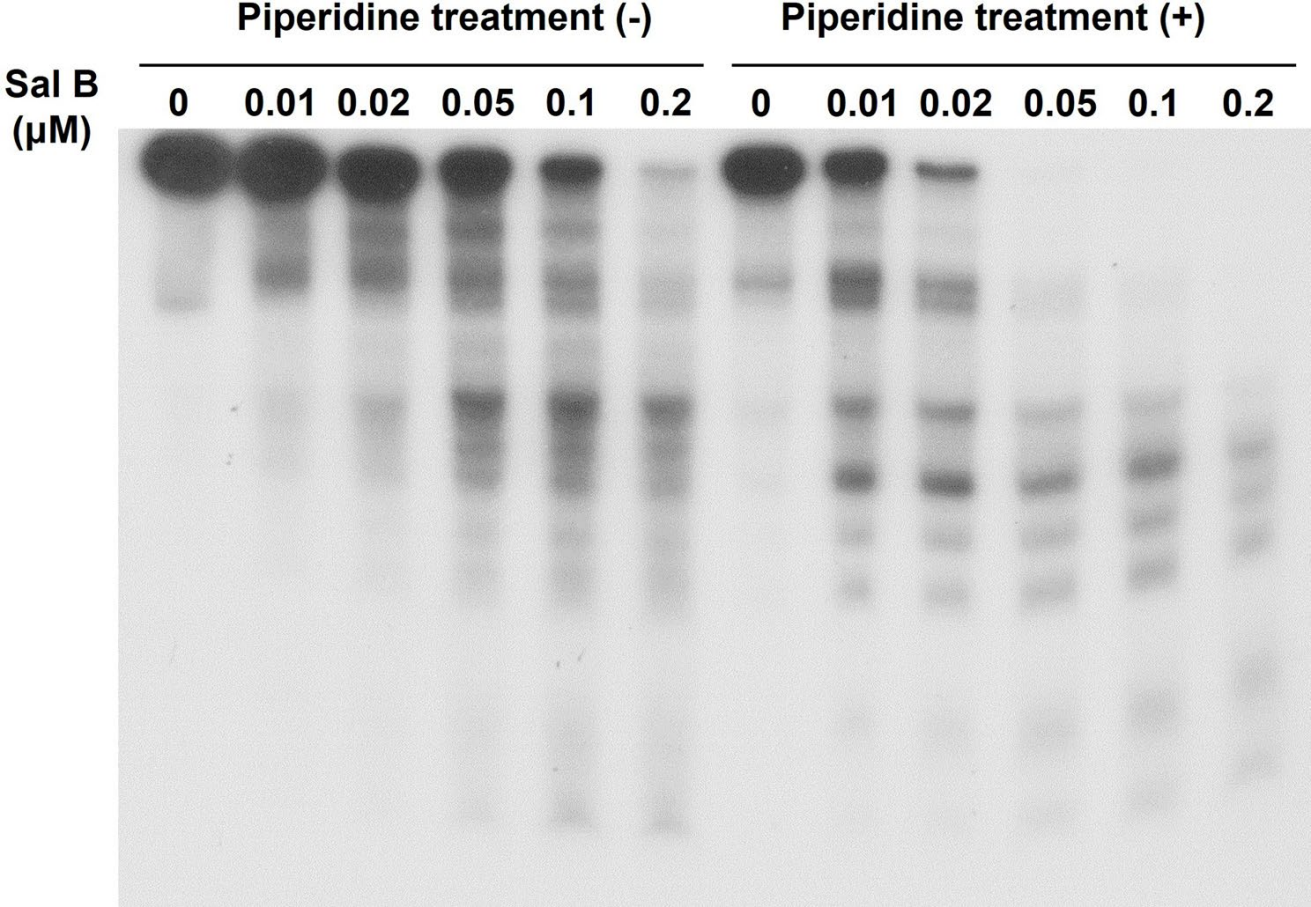
Autoradiogram of ^32^P-5′-end-labeled DNA fragments treated with Sal B in the presence of Cu(II) and NADH. The reaction mixtures contained the ^32^P-5′-end-labeled 309-bp fragment, 20 µM/base calf thymus DNA, 20 µM CuCl_2_, 100 µM NADH, and indicated concentrations of Sal B in 200 µL of 10 mM sodium phosphate buffer (pH 7.8) containing 5 µM DTPA. After incubation at 37 °C for 16 h, the DNA fragments were treated with or without hot piperidine and subjected to electrophoresis on a polyacrylamide gel

To gain insight into the mechanism of oxidative DNA damage induced by Sal B, we analyzed DNA damage using ^32^P-labeled DNA fragments. An autoradiogram of DNA fragments incubated with Sal B, Cu(II), and NADH, and subsequently treated with or without piperidine, is shown in Fig. 3. Oligonucleotides resulting from DNA cleavage were detected on this autoradiogram. In the presence of Cu(II) and NADH, Sal B cleaved DNA in a dose-dependent manner, and this was enhanced by piperidine treatment (Fig. 3). These results indicate that Sal B induced not only DNA backbone breakage but also modification of DNA bases, as piperidine cleaves DNA at sugars with modified bases. Furthermore, in the presence of Cu(II), H_2_O_2_ treatment led to DNA damage in ^32^P-labeled DNA like Sal B plus NADH (Supplemental Fig. 1).

### Site-specificity of DNA damage induced by Sal B in the presence of Cu(II) and NADH

**Fig. 4:**
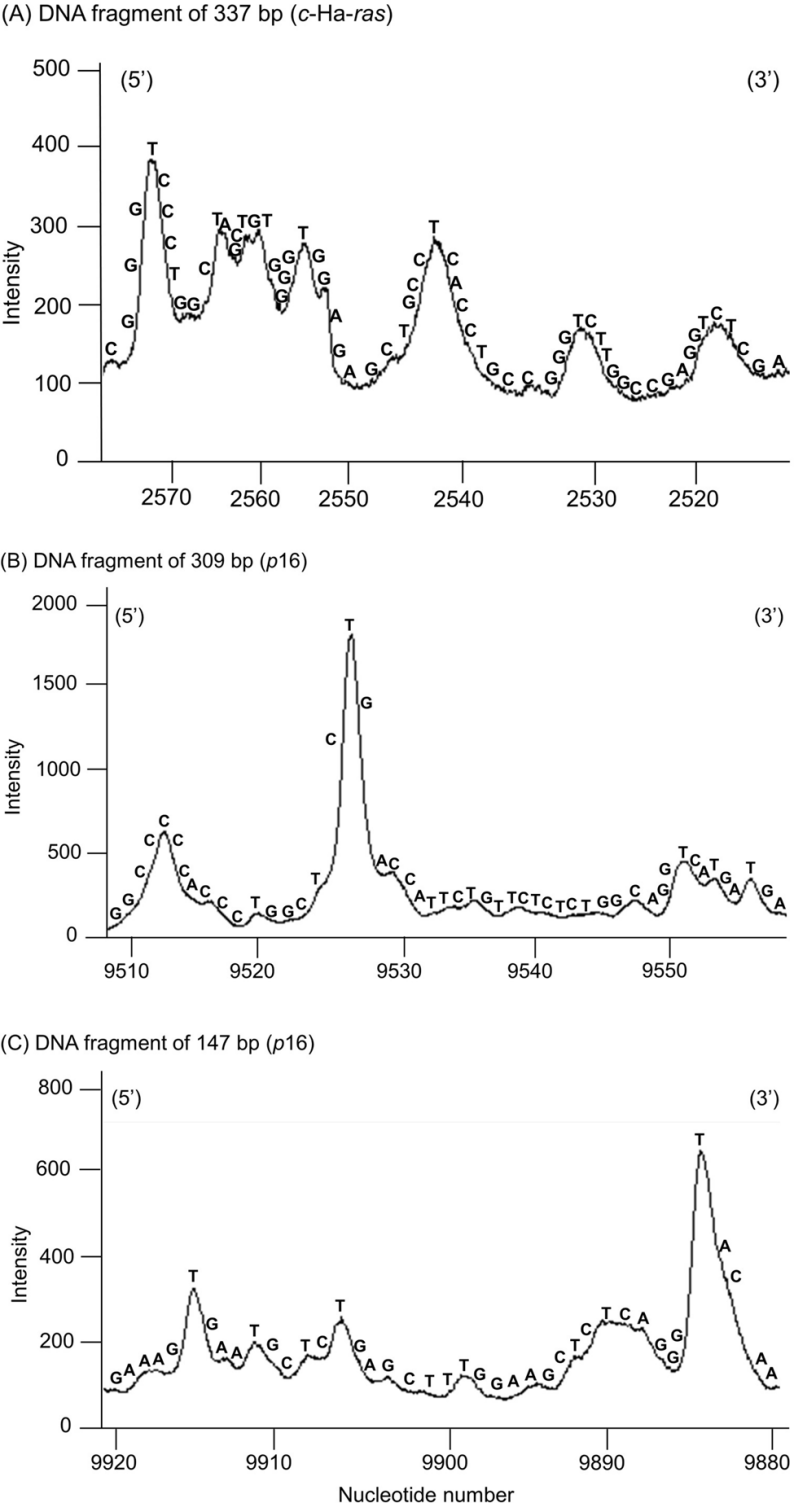
Site specificity of Sal B-induced DNA damage in ^32^P-5′-end-labeled DNA fragments. ^32^P-5′-end-labeled fragments of human c-Ha-*ras*-1 (337-bp [**A**]) and *p16* tumor suppressor gene (309-bp [**B**] or 147-bp [**C**]) were used. The reaction mixtures contained the ^32^P-5′-end-labeled fragment, 20 µM/base calf thymus DNA, 20 µM CuCl_2_, 100 µM NADH, and 0.05 µM Sal B in 200 µL of 10 mM sodium phosphate buffer (pH 7.8) containing 5 µM DTPA. After incubation at 37 ˚C for 16 h, the DNA fragments were treated with piperidine and subjected to electrophoresis on a polyacrylamide gel

The site-specificity of DNA damage induced by Sal B in combination with Cu(II) and NADH was determined by DNA sequencing using the Maxam-Gilbert procedure [28]. The relative intensity of DNA damage measured using a laser scanner is shown in Fig. 4. In the presence of Cu(II) and NADH, Sal B induced damage predominantly at thymine, especially located 5’ and/or 3’ to guanine, and some cytosine residues in DNA fragments obtained from the c-Ha-*ras* protooncogene (Fig. 4A) and human *p16* tumor suppressor genes (Fig. 4B and C). These base-specific DNA cleavages suggest the involvement of ROS other than the free hydroxyl radical (•OH) because •OH causes DNA cleavage with no preference for a specific cleavage site [11].

### Effects of ROS scavengers and a Cu(I) chelator on Sal B-induced DNA damage in the presence of Cu(II) and NADH

**Fig. 5:**
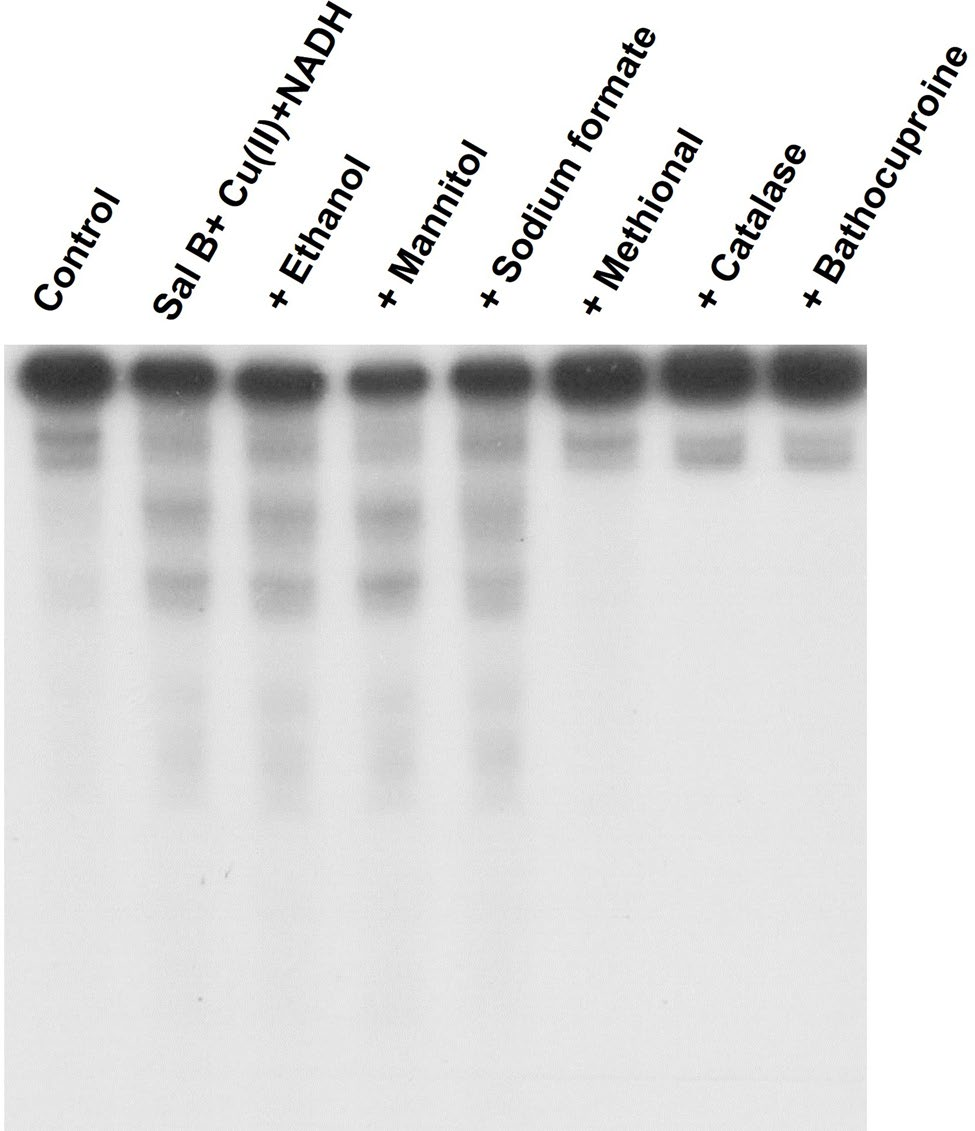
Effects of ROS scavengers and bathocuproine on Sal B-induced DNA damage in ^32^P-5′-end-labeled DNA fragments. The reaction mixtures contained the ^32^P-5′-end-labeled 147-bp fragment, 20 µM/base calf thymus DNA, 20 µM CuCl_2_, 100 µM NADH, 0.01 µM Sal B, and each scavenger or bathocuproine in 200 µL of 10 mM sodium phosphate buffer (pH 7.8) containing 5 µM DTPA. After incubation at 37 ˚C for 16 h, the DNA fragments were treated with piperidine and subjected to electrophoresis on a polyacrylamide gel. The concentrations of each scavenger and bathocuproine were as follows: 0.8 M ethanol, 0.1 M mannitol, 0.1 M sodium formate, 0.1 M methional, 30 U catalase, and 50 µM bathocuproine

To identify the species involved in the oxidative DNA damage induced by Sal B in combination with Cu(II) and NADH, we examined the effects of ROS scavengers and bathocuproine, a Cu(I)-specific chelator [29], on DNA damage (Fig. 5). Typical •OH scavengers such as ethanol, mannitol, and sodium formate did not prevent DNA damage. However, methional, which acts as a scavenger for a wide range of ROS [32], inhibited DNA damage, thus indicating involvement of ROS other than •OH. Bathocuproine and catalase, an H_2_O_2_ scavenger, also inhibited DNA damage. These results suggest that Cu(I) and H_2_O_2_ play important roles.

### HOMO localization of Sal B

**Fig. 6:**
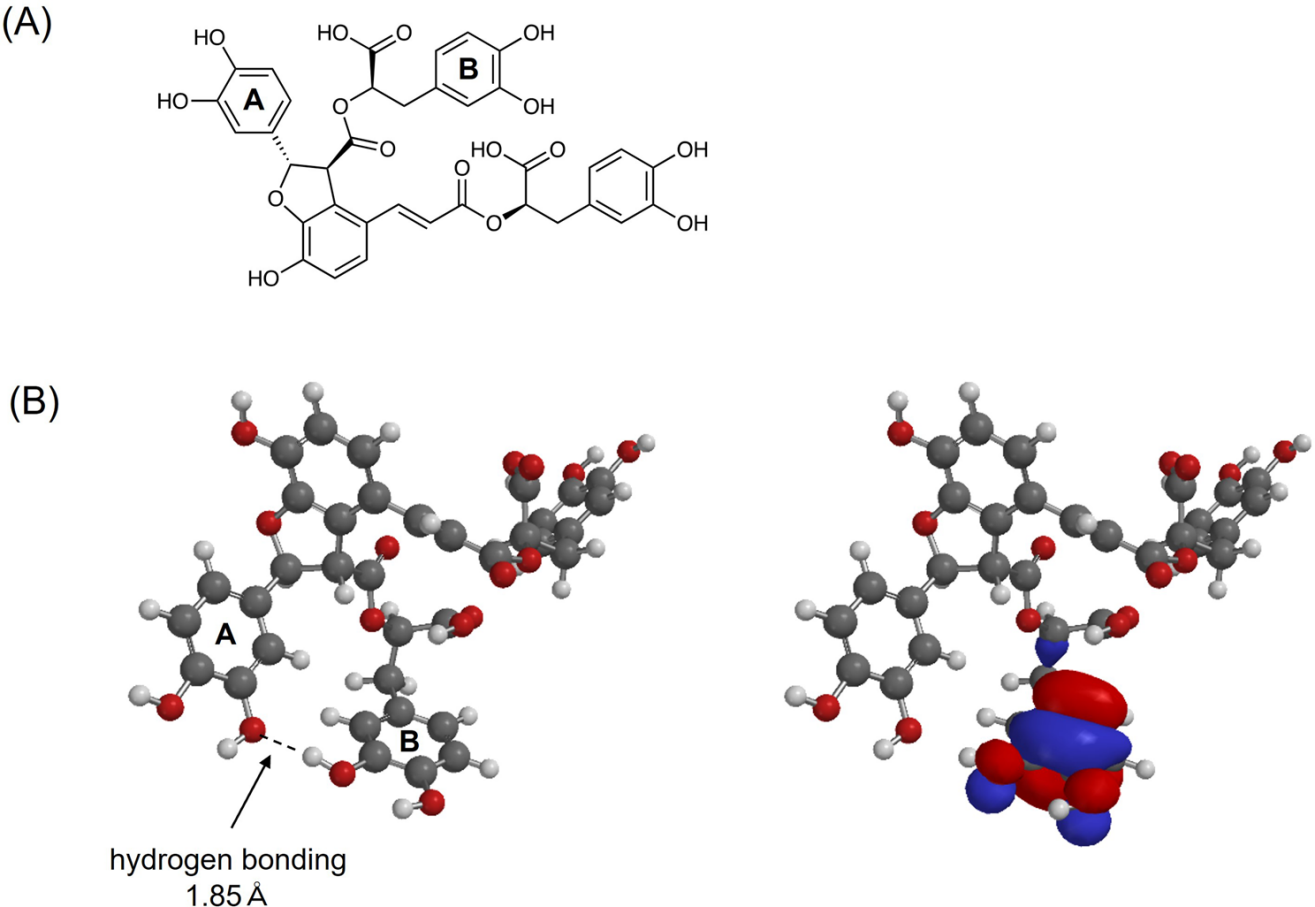
DFT-optimized structure and HOMO of Sal B. (**A**) Chemical structure of Sal B. (**B**) Three-dimensional chemical structure (optimized molecular structure) of Sal B. Geometry optimization was performed at the ωB97X-D/6-31G(d) level using Spartan 24. The left panel shows the most stable conformation of Sal B, while the right panel displays the electron density distribution of the HOMO, which is predominantly localized on one of the three catechol moieties

The generation of ROS via Cu(I)/Cu(II) redox cycling by polyphenols has been attributed to the presence of catechol moieties in their molecular frameworks [6]. Sal B contains three catechol units, prompting an investigation into which of these is primarily responsible for ROS generation (Fig. 6A). To this end, DFT calculations were performed to determine the most stable conformation of Sal B and to analyze the electron density distribution and energy level of the HOMO (Fig. 6B). The HOMO energy level of Sal B was calculated to be − 7.67 eV, and the HOMO was predominantly localized on one of the three catechol moieties. This localization appears to be stabilized by an intramolecular hydrogen bond, in which a hydroxyl group on the HOMO-localized catechol **B** acts as a hydrogen bond donor to the oxygen atom of a hydroxyl group on an adjacent catechol **A**. This intramolecular hydrogen-bonding interaction likely stabilizes the donating hydroxyl group and enhances the HOMO localization on the associated catechol moiety, thereby increasing the HOMO energy level in that region.

## Discussion

In this study, Sal B increased the level of 8-oxodG in HL-60 cells. 8-oxodG is reported to lead to G-to-T transversion via DNA misreplication [33, 34] and is related to carcinogenesis [35]. Treatment with bathocuproine, a Cu(I) chelator [29], inhibited 8-oxodG formation. In addition, 8-oxodG formation was not observed in HP100 cells, an H_2_O_2_-resistant clone of the HL-60 cell line [30]. These results indicate that Cu(I) and H_2_O_2_ play important roles in the formation of 8-oxodG. This is supported by the data indicating H_2_O_2_-induced DNA damage (Supplementary Fig. 1). However, in contrast to the lack of 8-oxodG observed in HP100 cells, bathocuproine did not completely inhibit 8-oxodG formation. These results suggest that Sal B may cause ROS generation in cells through mechanisms other than copper-mediated ROS generation. Indeed, studies have reported that the increase in ROS resulted from the Sal B-induced alteration of intracellular signaling pathways such as the AKT/mTOR and p38 pathways [9, 10]. Several studies have been reported on the intracellular uptake and metabolism of Sal B, which likely influence Sal B-induced oxidative DNA damage in cells. Sal B has been reported as a substrate of organic anion transporter 1B1, a hepatic uptake transporter [36]. However, there are few reports on the cellular uptake of Sal B in cells other than hepatocytes. Regarding the metabolism of Sal B, some pharmacokinetics studies showed that methylation is a major pathway in vivo [37, 38].

We also showed that treatment with Sal B and Cu(II) increased the level of 8-oxodG in calf thymus DNA. Notably, the formation of 8-oxodG induced by Sal B and Cu(II) was substantially enhanced by NADH. We have previously shown that NADH, an endogenous reductant [31], induced or enhanced metal-mediated DNA damage by establishing the redox cycle [11, 39]. According to our current results and previous studies, it is suggested that Sal B generates ROS and induces oxidative DNA damage via a Cu(I)/Cu(II) redox reaction, which could be enhanced by NADH at physiological concentrations.

To further elucidate the mechanisms underlying Sal B-induced oxidative DNA damage, we investigated DNA damage caused by Sal B in combination with Cu(II) and NADH using ^32^P-labeled DNA fragments from human cancer-related genes. The combination of Sal B, Cu(II), and NADH caused DNA cleavage, which was enhanced by treatment with piperidine, indicating that both base modifications and DNA strand breaks occurred. By employing the Maxam-Gilbert method, we showed that Sal B, Cu(II), and NADH caused site-specific damage to DNA, particularly at thymine adjacent to guanine. Considering that •OH typically induces non-specific DNA cleavage [11], the observed specificity of DNA damage suggests the involvement of ROS other than •OH. This was supported by our experiments using ROS scavengers. While typical •OH scavengers were ineffective, catalase (an H_2_O_2_ scavenger), bathocuproine (a Cu(I) chelator [29]), and methional (which scavenges multiple types of ROS other than •OH [32]) effectively prevented DNA damage. These findings suggest that a complex formed between H_2_O_2_ and Cu(I) plays a crucial role in inducing DNA damage. Since copper ion preferentially bind to guanine bases [40], Cu(I)-hydroperoxide complex could bind guanine. The complex may be considered to be a bound hydroxyl radical, which can release •OH and immediately attack an adjacent thymine residue, before it can be scavenged by •OH scavengers [41]. This is supported by findings that the Cu(II)-mediated oxidation initiated at guanine can propagate to adjacent thymine residues [42].

DFT calculations revealed that the HOMO of Sal B is predominantly localized on one of its three catechol moieties, indicating that this site likely serves as the primary electron donor. Notably, the hydroxyl group on this catechol **B** forms an intramolecular hydrogen bond, acting as a hydrogen bond donor to the oxygen atom of a hydroxyl group on an adjacent catechol **A**. This hydrogen-bonding interaction is presumed to stabilize the donating hydroxyl group, increase the local electron density, and thereby promote HOMO localization and elevate its energy level, resulting in enhanced reducing capability. Previously, we demonstrated that rosmarinic acid, a structurally related polyphenol bearing two catechol units, also generates ROS through Cu(I)/Cu(II)-mediated redox cycling and induces DNA cleavage activity [13]. However, in contrast to Sal B, rosmarinic acid does not form an intramolecular hydrogen bond between its catechol groups, and its calculated HOMO energy (-7.81 eV) is lower than that of Sal B (-7.67 eV). These findings suggest that intramolecular hydrogen bonding in Sal B plays a crucial role in increasing the HOMO energy and electron-donating ability. Taken together, these results indicate that the catechol moiety bearing the localized HOMO is the redox-active site responsible for initiating Cu(II) reduction.

**Fig. 7:**
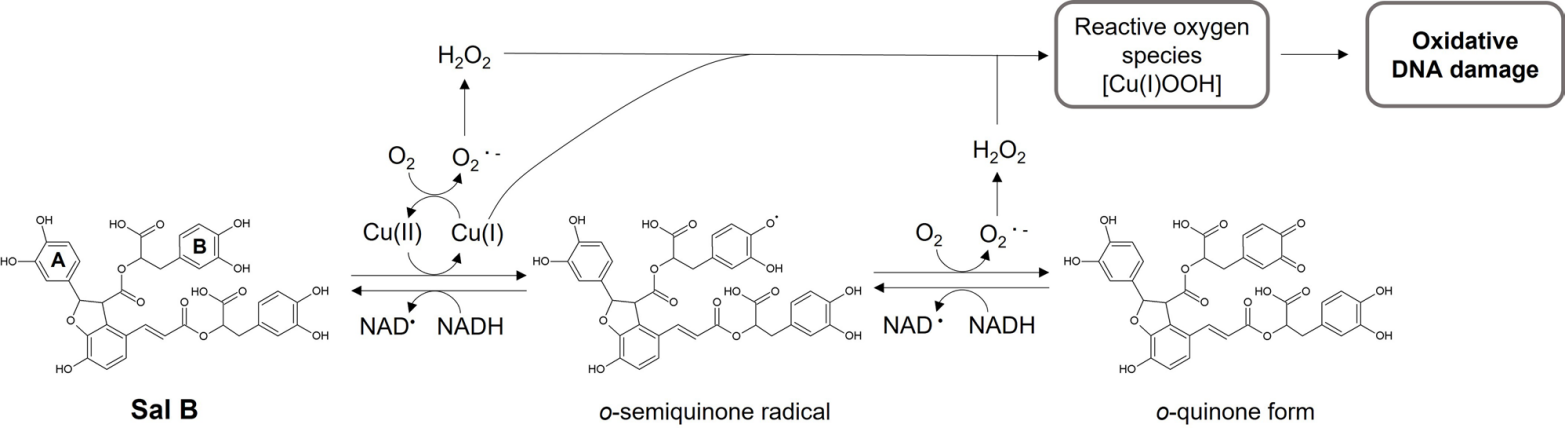
A possible mechanism of Cu(II) and NADH-mediated ROS generation and DNA damage induced by Sal B

Taking into account these results collectively, a proposed mechanism for the oxidative DNA damage induced by Sal B in the presence of Cu(II) and NADH is illustrated in Fig. 7. The electron-rich catechol group **B** is susceptible to autoxidation, resulting in the formation of the corresponding *o*-semiquinone radical and *o*-quinone. Concomitantly, Cu(II) is reduced to Cu(I), which facilitates the generation of superoxide anion (O₂^•^⁻) from molecular oxygen and its subsequent conversion to H₂O₂. The interaction between Cu(I) and H₂O₂ likely generates a Cu(I)-hydroperoxide complex, which serves as a key reactive species responsible for oxidative DNA cleavage. The *o*-quinone radical and *o*-semiquinone intermediates can be reduced back to Sal B by NADH, establishing a redox cycle that amplifies ROS production and promotes sustained DNA damage.

## Conclusions

In the present study, we demonstrated that Sal B caused copper-dependent ROS generation, resulting in oxidative DNA damage. This process was markedly enhanced by NADH. This is proposed as another possible underlying mechanism of Sal B-induced ROS generation, in addition to those reported in previous studies [9, 10]. The oxidative DNA damage was caused by Sal B in the presence of physiologically relevant concentrations of Cu(II) (20 µM) [43] and NADH (100 µM) [44], raising the possibility that Sal B administration could induce oxidative damage DNA in vivo. This study highlights the need for a careful risk assessment of Sal B when used as a therapeutic agent.

## Supplementary Information

Below is the link to the electronic supplementary material.

Supplementary Material 1

## Data Availability

No datasets were generated or analysed during the current study.

## Abbreviations

Sal B: Salvianolic acid B
ROS: Reactive oxygen species
8-oxodG: 8-oxo-7,8-dihydro-2’-deoxyguanosine
DTPA: Diethylenetriamine-N, N,N’,N”,N”-pentaacetic acid
HPLC: High-performance liquid chromatography
ECD: Electrochemical detector
HOMO: Highest occupied molecular orbital
DFT: Density functional theory
SD: Standard deviation
