# Supplementary material for: The Chinese herb component salvianolic acid B induces copper-mediated reactive oxygen species generation and oxidative DNA damage
Source: Genes Environ. 2025 Oct 30;47:20. doi: 10.1186/s41021-025-00344-w (PMC12574035; doi:10.1186/s41021-025-00344-w)
Supplement: Supplementary file 1 — Supplementary Material 1 [file 41021_2025_344_MOESM1_ESM.pdf]

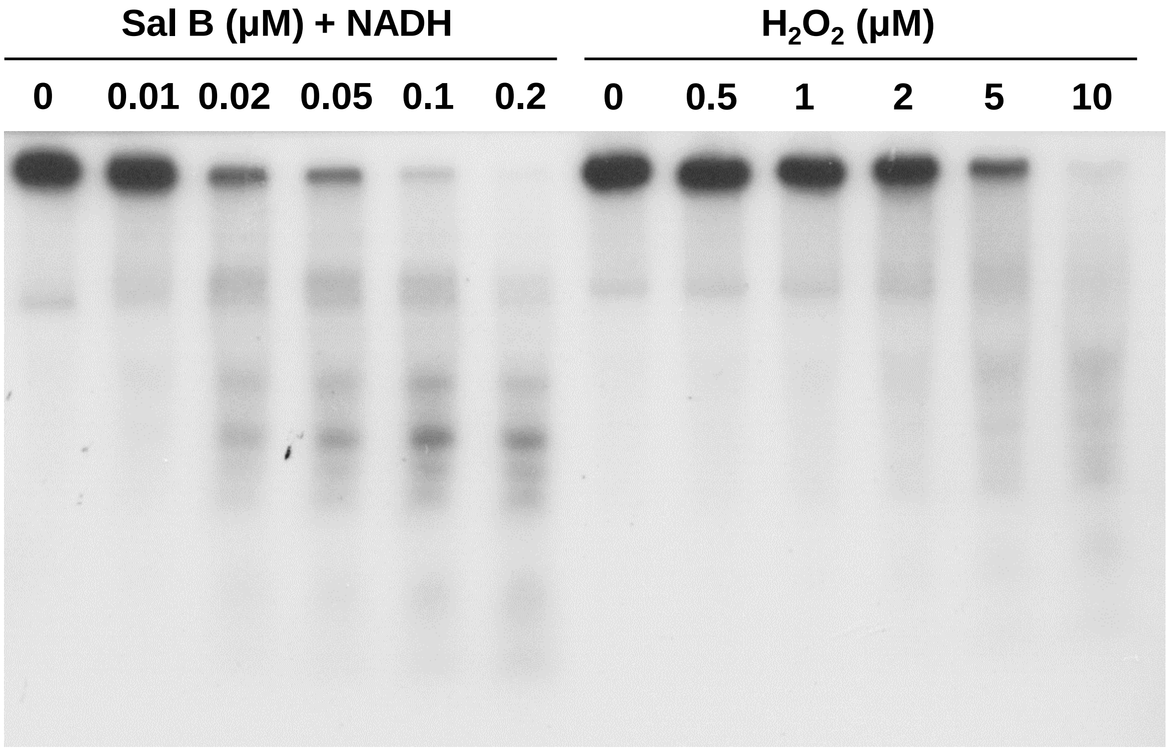

Supplemental Figure 1. Autoradiogram of ^{32}P-5′-end-labeled DNA fragments treated with Sal B plus NADH or H_2O_2 in the presence of Cu(II). The reaction mixtures contained the ^{32}P-5′-end-labeled 309-bp fragment, 20 μM/base calf thymus DNA, 20 μM $CuCl_2$, 100 μM NADH, and indicated concentrations of Sal B and H_2O_2 in 200 μL of 10 mM sodium phosphate buffer (pH 7.8) containing 5 μM DTPA. After incubation at 37 ºC for 16 h, the DNA fragments were treated with hot piperidine and subjected to electrophoresis on a polyacrylamide gel.
